# Mechanisms Underlying the Anti-Tumoral Effects of *Citrus bergamia* Juice

**DOI:** 10.1371/journal.pone.0061484

**Published:** 2013-04-16

**Authors:** Simona Delle Monache, Patrizia Sanità, Elena Trapasso, Maria Rita Ursino, Paola Dugo, Marina Russo, Nadia Ferlazzo, Gioacchino Calapai, Adriano Angelucci, Michele Navarra

**Affiliations:** 1 Department of Biotechnological and Applied Clinical Sciences, University of L’Aquila, L’Aquila, Italy; 2 Department of Drug Sciences and Health Products, University of Messina, Messina, Italy; 3 Department of Clinical and Experimental Medicine, University of Messina, Messina, Italy; 4 Istituto Di Ricovero e Cura a Carattere Scientifico centro neurolesi “Bonino-Pulejo”, Messina, Italy; Enzo Life Sciences, Inc., United States of America

## Abstract

Based on the growing deal of data concerning the biological activity of flavonoid-rich natural products, the aim of the present study was to explore *in vitro* the potential anti-tumoral activity of *Citrus Bergamia* (bergamot) juice (BJ), determining its molecular interaction with cancer cells. Here we show that BJ reduced growth rate of different cancer cell lines, with the maximal growth inhibition observed in neuroblastoma cells (SH-SY5Y) after 72 hs of exposure to 5% BJ. The SH-SY5Y antiproliferative effect elicited by BJ was not due to a cytotoxic action and it did not induce apoptosis. Instead, BJ stimulated the arrest in the G1 phase of cell cycle and determined a modification in cellular morphology, causing a marked increase of detached cells. The inhibition of adhesive capacity on different physiologic substrates and on endothelial cells monolayer were correlated with an impairment of actin filaments, a reduction in the expression of the active form of focal adhesion kinase (FAK) that in turn caused inhibition of cell migration. In parallel, BJ seemed to hinder the association between the neural cell adhesion molecule (NCAM) and FAK. Our data suggest a mechanisms through which BJ can inhibit important molecular pathways related to cancer-associated aggressive phenotype and offer new suggestions for further studies on the role of BJ in cancer treatment.

## Introduction

*Citrus Bergamia* Risso & Poiteau, a small tree belonging to the *Rutaceae* family, is cultivated almost exclusively along the southern coast of Calabria region (Italy), where the particular environmental conditions are favourable for its cultivation. Bergamot fruit is mostly used for the extraction of essential oil, widely used in perfume industry and recently investigated for its beneficial effects in neuroprotection [1]. Bergamot juice (BJ), instead, obtained from the endocarp of the fruit, is considered just a secondary and discarded product. Different studies have analyzed the chemical composition of BJ [2], [3], [4], [5] revealing its elevated content in flavonoids most of which can exert beneficial effect on human health. The most recurrent flavonoids present in BJ include flavanones and flavones.

Inhibition of carcinogenesis by flavonoids has been demonstrated both *in vitro* and *in vivo* [6]. Several underlying mechanisms have been proposed, including the suppression of cyclooxygenase-2 (COX-2) expression [7], decrease of reactive oxygen species (ROS) [8], [9], modulation of oncogenic signalling pathways and down-regulation of nuclear transcription factor kappa B (NF-kB) [10], [11]. The resulting effects are the arrest of proliferation and the induction of the apoptosis in cancer cells [12], [13]. Moreover, several flavonoids have demonstrated radical-scavenging and anti-inflammatory activities [14], [15], [16]. Importantly, flavonoids do not present any significant toxicological risk and the safety margin for their therapeutic use in humans is very large [17]. In addition, other studies suggest that flavonoids could inhibit tumoral invasion and metastasis [18], [19], [20]. Findings in animals and investigations by using different cellular models suggest that certain flavonoids could effectively inhibit also tumour progression. In particular, naringin, one of the flavonoids present in the BJ, was able to reduce the high glucose-induced ICAM-1 expression via the p38 MAPK signalling pathway, contributing to the inhibition of monocyte adhesion to endothelial cells [19]. Moreover it has been demonstrated that flavanone and 2′-OH flavanone perturb the invasion and metastasis of lung cancer cells probably through inactivation of ERK 1/2 and p38^MAPK^ signalling pathways [20]. Much of these effects can be attributable to the ability of flavonoids to interact with mitogenic or migratory signalling pathways but the precise underlying molecular mechanisms remain largely unclear.

The antitumor potential of natural products rich in flavonoids is probably underestimated and further studies elucidating their molecular interaction are warranted. In this study we demonstrate that BJ has a potential anti-tumoral capacity and that in a neuroblastoma cell model this action is realized mainly through an early impairment in cell adhesive and migratory machinery.

## Results

### Flavonoids Composition of Bergamot Juice

**Figure 1:**
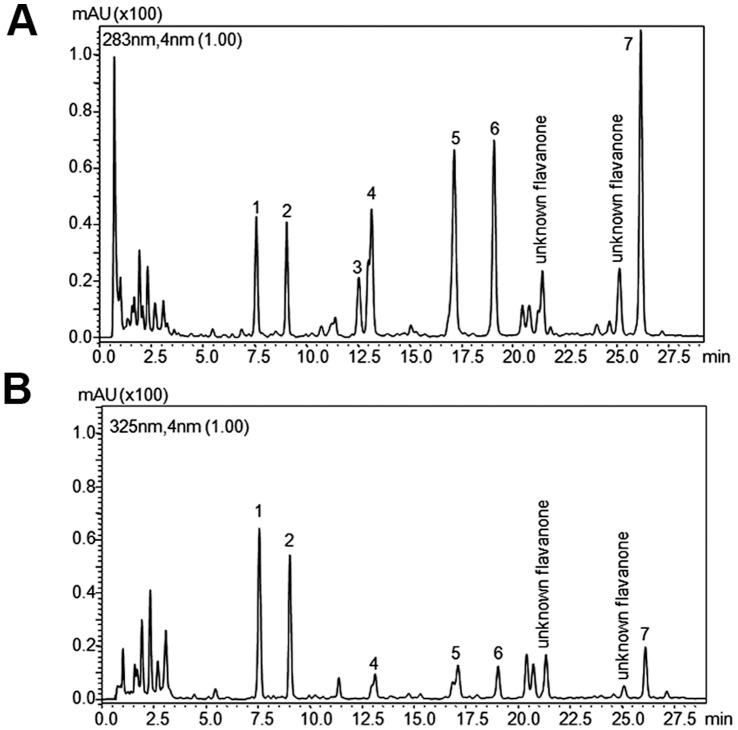
RP-HPLC chromatograms of BJ. The analysis was performed at two different wavelengths, 283 (A) and 325 (B) nm. The sample was examined for five times and a representative chromatogram is shown. For peak identification see Table 1.

Figure 1 shows the chromatograms obtained for the BJ used in our study and extracted at 283 nm and 325 nm. BJ is characterized by the presence of seven flavanones showing maximum absorption at 283 nm (Fig. 1A) namely eriocitrin ([M-H]- 595 (287)), neoeriocitrin ([M-H]- 595), narirutin ([M-H]- 595 (271)), naringin ([M-H]- 595), neohesperidin ([M-H]- 609), naringin-di-oxalate ([M-H]- 621 (579)) neohesperidin-di-oxalate ([M-H]- 651 (609)), and five flavones that show maximum absorption at 325 nm (Fig. 1B): apigenin-6,8-di-C-glucoside ([M-H]- 593), diosmetin-6,8-di-C-glucoside ([M-H]- 623), neodiosmin ([M-H]- 607), rhoifolin ([M-H]- 577), diosmin ([M-H]- 607 (299)). These qualitative data are in good agreement with those previously reported in literature [2], [3], [4], [5] and confirmed that BJ is rich in flavonoids. Table 1 summarizes the flavonoid content (mg/L ± SEM) of the sample, and also illustrates the maximum absorption wavelength (λ_max_), parent ion ([M-H]^-^) and coefficient of variation (CV%) values of each compound in the sample analyzed. Neoeriocitrin, naringin and neohesperidin are the most abundant flavanones in the BJ analyzed, representing with naringin-di-oxalate and neohesperidin-di-oxalate about the 90% of total flavanone-*O*-glycosides. Our data, concerning the quantitative content of flavanone-*β*-neohesperidosides of BJ, are in agreement with previous reports [5]. Identification was carried out by combining the information of both UV and MS data with that of reference materials when available.

**Table 1:** Concentration (mg/L) of flavonoids in bergamot juice.

N°	Compounds	Bergamot juice
1	Apigenin-6,8-di-*C*-glucoside	n.a.*
2	Diosmetin-6,8-di-*C*-glucoside	n.a.*
3	Crisoeriol-7-*O*-neohesperidoside-4′-glucoside	n.a.*
4	Eriocitrin	85
5	Neoeriocitrin	115
6	Naringin	84
7	Neohesperidin	108

* n.a.: standard was not available in sufficient amount for quantitative calculation.

### Effects of BJ on Cell Growth

**Figure 2:**
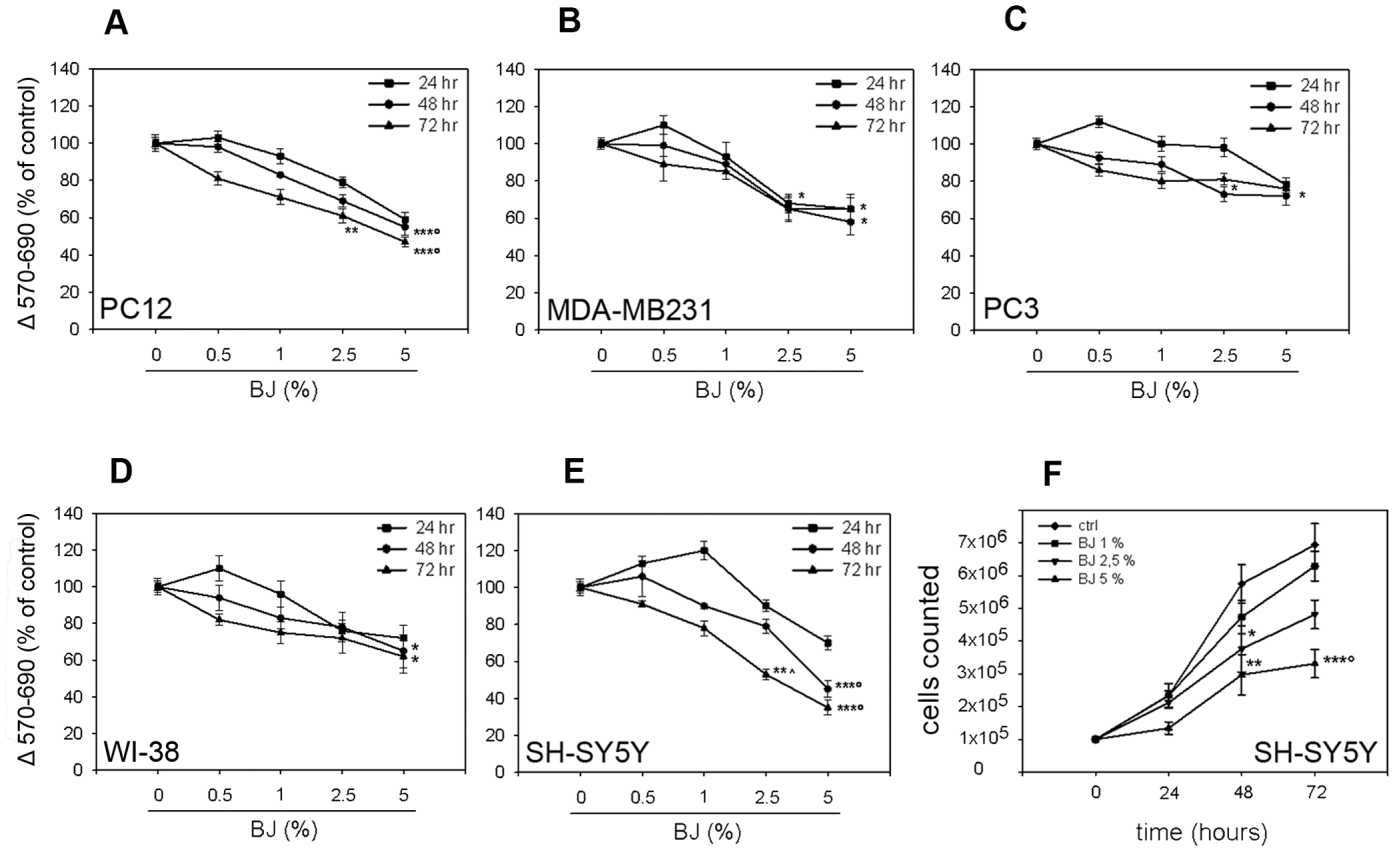
Effect of BJ on cell proliferation. PC-12 (A), MDA-MB231 (B), PC3 (C), WI-38 (D) and SH-SY5Y (E) cells were incubated with bergamot juice (from 0.5 to 5%) for 24, 48 and 72 hs and assayed by MTT test. Results are expressed as percentage of absorbance respect to control cells (100%). Analysis of the SH-SY5Y proliferation was performed also by cell count assays (F). Experimental data showed that, although with different extent, BJ reduced growth rate of several cell lines, with the maximal effect in the SH-SY5Y. The results are expressed as means ± SEM from at least three independent experiments performed in eightplicate (MTT test) or in triplicate (cell counting). *P<0.05 *vs* ctrl; **P<0.01 *vs* ctrl; ***P<0.001 *vs* ctrl, BJ 0.5 and 1%; °P<0.05 *vs* BJ 2.5%; ^∧^P<0.05 *vs* BJ 1%.

In order to evaluate the anticancer properties of BJ, first we checked its anti-proliferative activity on different cancer cell lines by MTT assay. As shown in figure 2, incubation of PC3, MDA-MB231, PC12 and SH-SY5Y cells with increasing concentration of BJ ranging from 0.5% to 5% for 24, 48 and 72 hs, reduced proliferation in all cell lines with a similar trend. A significant decrease of growth rate with a peak value of 52±3% (P<0.001 *vs* untreated cells; Fig. 2A) was demonstrated in PC12 cells after 72 hs of BJ incubation, while the 35% and 15% of inhibition in cell proliferation were observed in MDA-MB231 and PC3 cells, respectively (Fig. 2B and 2C). The greatest inhibitory effect was reported in SH-SY5Y cells in which was observed a time- and concentration-dependent reduction in cell growth, reaching the maximal extent (65±4%) after 72 hs of exposure to BJ 5% (P<0.001 *vs* untreated cultures; Fig. 2E). The results obtained by MTT analysis in SH-SY5Y cells were confirmed by the cell count assay (Fig. 2F). However, also the WI-38 diploid fibroblasts cell line showed a slight inhibition of the proliferation rate (Fig. 2D).

### Mechanisms Underlying the Antiproliferative Effects of BJ

**Figure 3:**
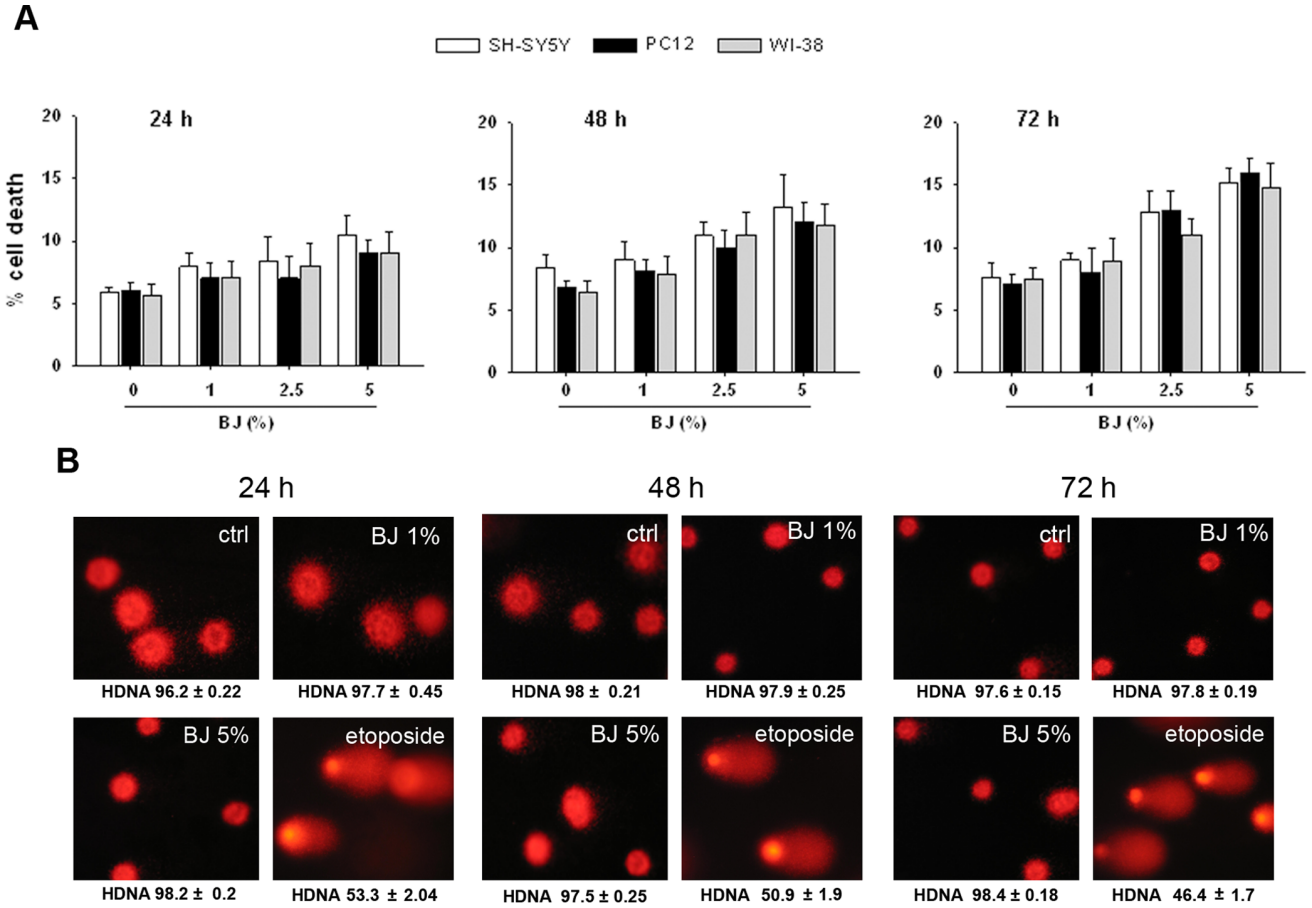
Cytotoxic effect of BJ. (A) Cytotoxic action of increasing concentrations of BJ (1–5%) was determined in SH-SY5Y, PC12 and WI-38 cells by trypan blue dye exclusion test. The assays were performed for 24, 48 and 72 hs and expressed as % of cell death. Data are the mean ± SEM of three independent experiments. Results display that BJ did not induced cytotoxicity neither in normal nor in tumoral cells. (B) Assessment of DNA damage in SH-SY5Y cells exposed to BJ performed by comet assay. In the panel are reported the images captured by fluorescence microscopy after 24 (on the left), 48 (in the middle) and 72 hs (on the right) of treatment. A representative experiment that was replicated three times with similar results is shown. The images display the round and intact nucleus observed in both BJ-treated and untreated cells, suggesting the lack of genotoxicity by BJ. HDNA: % head DNA. Nuclei were visualized by fluorescence microscopy at a magnification of 400x.

In order to detect eventual cytotoxic effect of BJ, the cell lines in which was observed the greatest growth inhibition (SH-SY5Y and PC12 cells) were exposed to different concentrations of BJ (1–5%) for 24–72 hs, and then the trypan blue dye exclusion assay was used to detect dead cells. As comparison, diploid fibroblasts WI-38 cells, were used. Figure 3A shows that BJ did not induce significant increase in cell death neither in SH-SY5Y cells nor in PC12 or in WI-38 cells (Fig. 3A). Moreover, results of comet assay suggested that BJ at concentration ranging from 1 to 5% for 24–72 hs of incubation did not induce SH-SY5Y DNA damage (Fig. 3B). Cell parameters from 100 individual cells were recorded and analyzed for comparative data between BJ-treated and untreated cultures (see materials and methods) without obtaining significant differences (data not shown).

**Figure 4:**
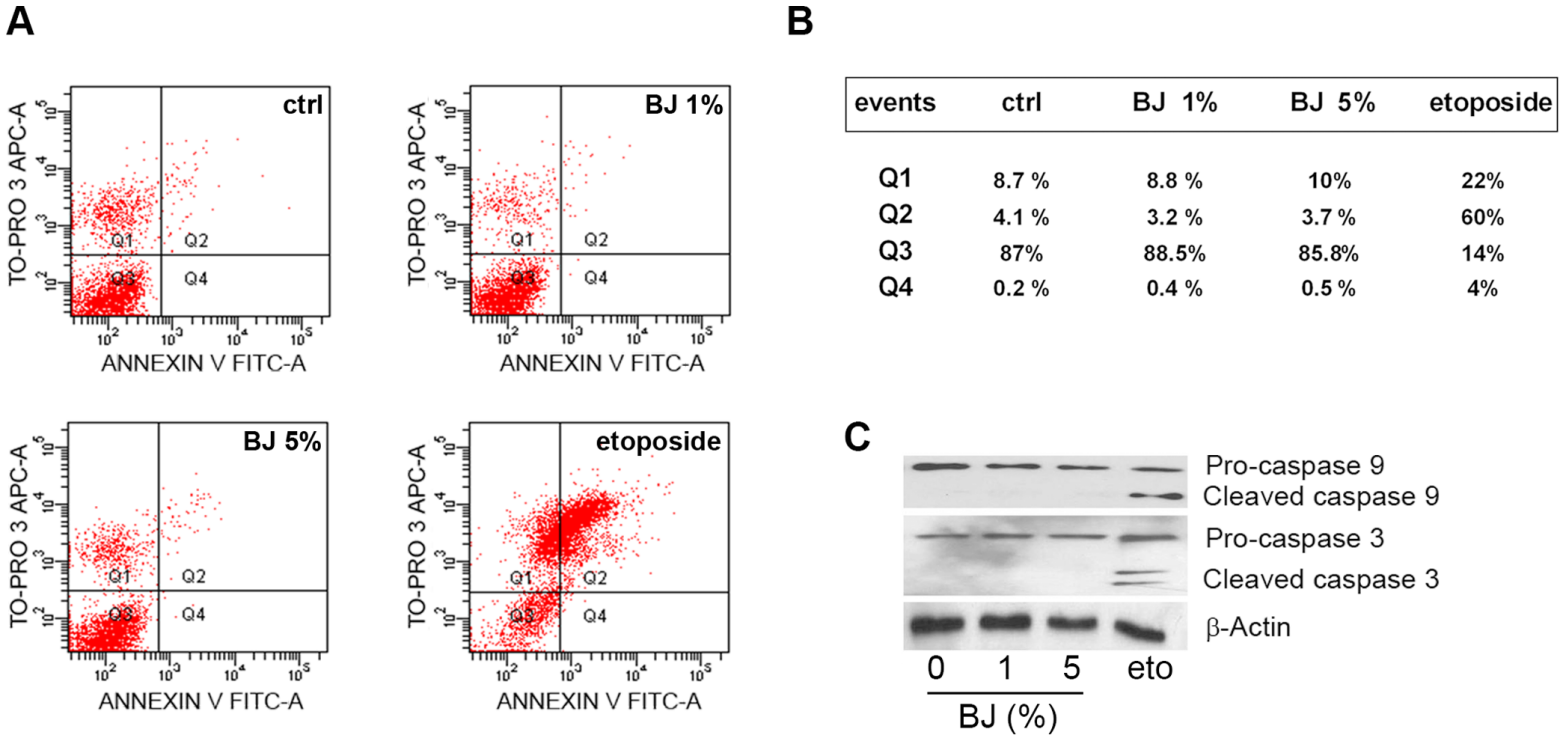
Evaluation of apoptosis on the SH-SY5Y cells exposed to BJ. (A) Cytofluorimetric evaluation of apoptosis; representative Annexin V *versus* PI dot plot analyses of SH-SY5Y cells treated with BJ for 72 hs. (B) Mean percentage of events in each quarter (Q) of cytofluorimetric analyses: Q3 contains the vital cells, Q4 the cells in early apoptosis, Q2 the cells in late apoptosis and Q1 the necrotic cells. The FACS analysis presented is representative of three different experiments. (C) Analysis of caspase-3 and caspase-9 expression in SH-SY5Y cells treated for 72 hs with BJ or etoposide (eto). Lower molecular weight bands of caspases represent the active form. Data show the lack of apoptosis after 72 hs of exposure to BJ.

Furthermore, the annexin V staining assay performed in SH-SY5Y cells demonstrated that BJ was unable to activate the programmed cell death. Indeed dot-plot in figure 4A indicates the lack of apoptosis after 72 hs of exposure to 1–5% BJ. The same situation was observed treating SH-SY5Y cells for 6, 24 and 48 hs (data not shown). On the contrary, the well-known pro-apoptotic drug etoposide, used as positive control, caused an important increase in apoptotic cells (Fig. 4A and B). Failure in the detection of cleaved caspase-3 and 9 in the SH-SY5Y exposed to BJ, confirmed the absence of apoptosis (Fig. 4C).

**Figure 5:**
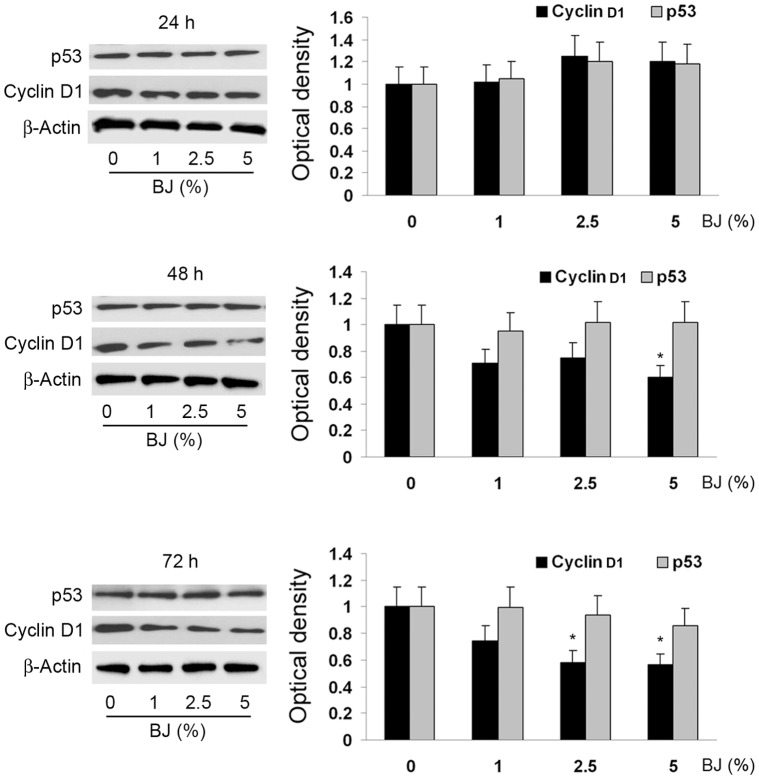
Analysis of p53 and cyclin D1 expression in SH-SY5Y treated with BJ. On the left of the panel are display the immunoblot of SH-SY5Y cells exposed to 1–5% BJ for 24–72 hs: a representative of three separate experiments is shown. On the right of the panel are presented the quantification ± SEM of both cyclin D1 and p53 expressions from three independent blots performed after 24–72 hs of BJ incubation. Autoradiographic bands were quantified by ImageJ software and normalized for β-actin levels. Data are extrapolated in reference of the values detected in control cells, which are arbitrarily assigned as 1. Results of Western blot documented that BJ decreases the expression of cyclin D1, unchanging the level of p53. *P<0.05 *vs* ctrl.

To further elucidate the mechanisms by which BJ exerted its antiproliferative activity, we examined the cell cycle progression of SH-SY5Y by cytofluorimetric analysis. BJ reduced the G2/M phase in a concentration- and time-dependent manner, up to its complete disappearance following 72 hs of SH-SY5Y exposure at the highest BJ concentrations (Table 2). In parallel we observed the increase of the cells in the G1 phase. In order to evaluate the involvement of important cell cycle regulators, Western blot analysis of p53 and cyclin D1 was performed. Figure 5 shows that BJ did not change the level of p53, but decreased the expression of cyclin D1 in a time-dependent trend, with the maximal reduction detected after 72 hs of treatment with the BJ 2.5 and 5% (P<0.05 *vs* untreated cultures). Since cyclin D1 governs the transit through the G1 phase of the cell cycle, we suggest a correlation between its reduced expression, the cell cycle arrest and the inhibition of cellular proliferation.

**Table 2:** BJ effect on cell cycle progression.

Cycle phase	Control	BJ 1%	BJ 2.5%	BJ 5%
		***24 hours treatment***		
G1	60±5.4%	64±5.1%	72±6.6%	78±7.4%
S	26±2.3%	22±1.1%	16±1.6%	12±1.9%
G2/M	14±1.1%	14±0.9%	12±1.1%	10±1.1%
		***48 hours treatment***		
G1	58±6.2%	64±7.4%	76±7.6%	80±8.0%
S	30±3.1%	24±2.4%	16±2.2%	14±2.6%
G2/M	12±0.9%	12±1.2%	8±0.8%	6±0.9%
		***72 hours treatment***		
G1	62±5.8%	74±4.9%	80±7.5%	88±9.1%
S	28±2.3%	19±1.8%	16±1.8%	12±2.1%
G2/M	10±0.6%	7±0.9%	4±0.8%	0±0.6%

Results show that BJ arrests cell cycle at G1 phase, reducing S and G2/M phases in time and concentration dependent manner. Data are represented as percentages of the total cell population and are the mean ± SEM of five independent experiments.

### Anti-adhesive Effect of BJ

**Figure 6:**
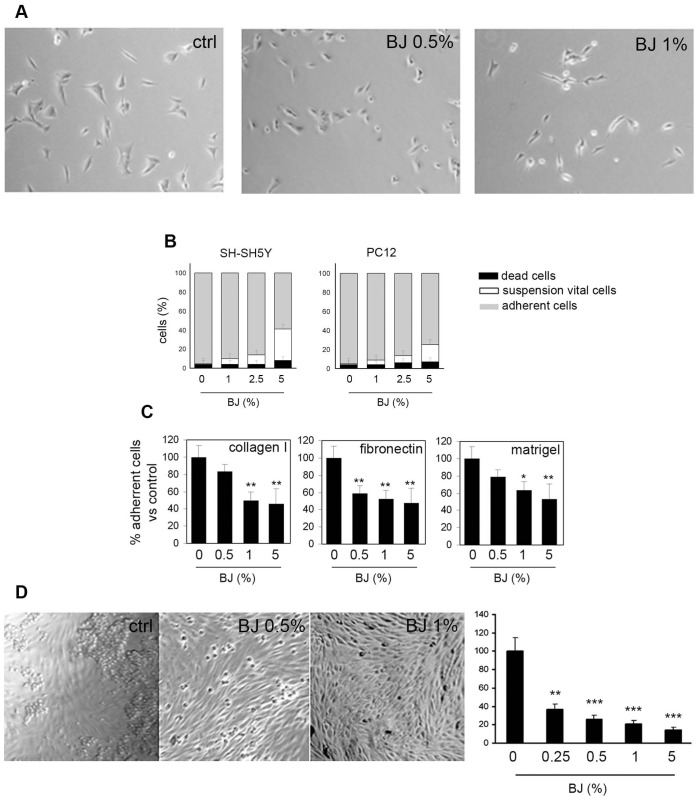
Adhesion inhibition by BJ treatment. (A) Representative images showing the decreased adhesive capacity of SH-SY5Y cells following 2 hs of BJ treatment. Cells were visualized by optical microscopy at a magnification of 200x. (B) Adhesion assay performed by plating SH-SY5Y and PC12 cells on non-coated plastic surface for 24 hs. Data were expressed as percentage of the total cells present in the well and are the mean ± SEM of three different experiments. (C) Number of adhered cells obtained after plating SH-SY5Y cells on three different physiological substrates (Collagen I, Fibronectin and Matrigel) in presence of BJ for 24 hs. The values are expressed as mean percentage with respect to untreated cultures of three different experiments (± SEM). (D) Adhesive skill of SH-SY5Y cells treated with BJ at 0.5 and 1% in comparison with untreated cells when seeded on HUVEC monolayers. Representative images show the decrease capability of SH-SY5Y cell (see the number of round cells) to join to HUVEC cells. Values obtained by counting attached SH-SY5Y cells are shown in the histogram. Data are expressed as mean ± SEM counts of five different optical fields. *P<0.05; **P<0.01; ***P<0.001 *vs* control. Collectively the results demonstrated that BJ impaired cells adhesiveness.

It is well known that cell adhesion plays a crucial role in tumor invasion and metastasis formation. To this end we investigated the ability of BJ to alter the cell adhesion on various substrates. As illustrated in figure 6A, 2 hs of exposure with BJ altered the SH-SY5Y cell morphology, and the cells acquired a round shape causing a marked reduction of cell adhesion to the plastic surface. A similar behavior was observed also in PC12 cells (data not shown). After 24 hs of incubation with various dilutions of BJ either SH-SY5Y and PC12 cells were collected by gentle agitation and counted in the presence of trypan blue. Afterwards, still attached cells were detached by trypsin and counted. The results clearly show that BJ decreased cell adhesiveness in a concentration-dependent manner (Fig. 6B). Trypan blue staining revealed that the cell in suspension were mostly vital. The marked anti-adhesive activity of the BJ was registered especially in SH-SY5Y cells and remained almost unchanged throughout 48 and 72 hs of BJ treatment (data not shown). Therefore, specific adhesion assays were performed only with SH-SY5Y cells (Fig. 6C). The cells were treated with BJ at increasing concentrations ranging from 0.5 to 5% for 24 hs and then seeded on different physiological substrates like collagen I, fibronectin or matrigel. In all experimental conditions we observed a significant decrease of adhesive capacity in a dose-dependent manner. The highest inhibitory effect was recorded in presence of fibronectin, when cell adhesion was markedly reduced also by the lowest BJ concentration (P<0.01 *vs* untreated cultures; Fig. 6C).

We also evaluated the adhesive ability of the SH-SY5Y cells exposed to the BJ for 24 hs on HUVEC monolayer, that appeared about 3 fold lower than untreated cells already in presence of 0.25% BJ (Fig. 6D). Interestingly, we observed that untreated cells grouped together forming clusters respect to BJ treated cells, suggesting that BJ as well as reducing adhesion ability of SH-SY5Y on HUVEC monolayer may interfere with the formation of cell-cell contacts.

### BJ Impairs Migration of SH-SY5Y Cells and Interferes with Actin Polymerization

**Figure 7:**
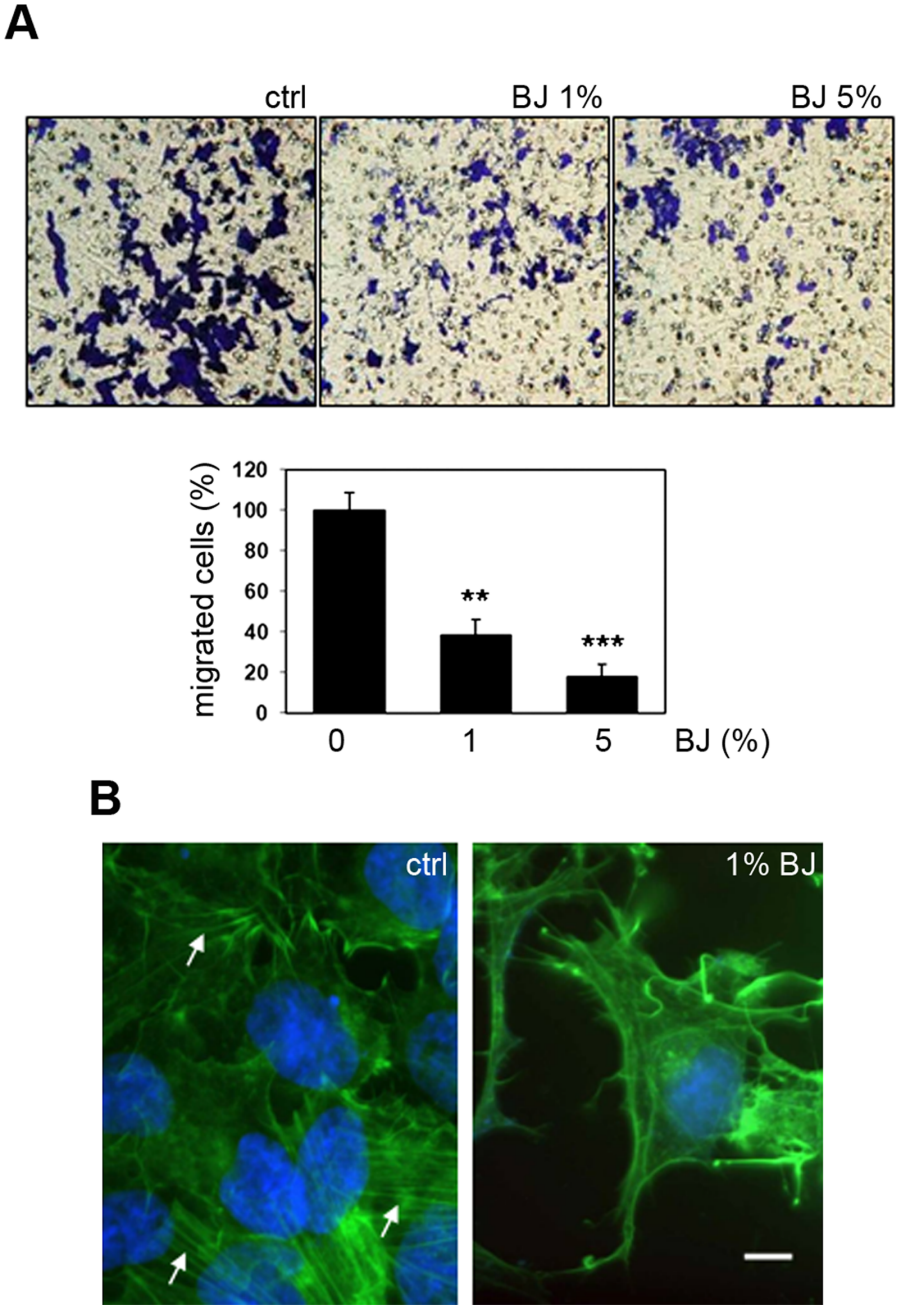
BJ impairs SH-SY5Y migration interfering with actin polymerization. (A) Effect of BJ on SH-SY5Y migration through gelatine coated filters after 24 hs of treatment. In the upper panel representative images of coated-filters are shown. The graphic in the lower ones represents the mean ± SEM of the migrated cells, as described in the material and methods. **P<0.01 and ***P<0.001 *vs* control. (B) Fluorescence F-actin labelling of SH-SY5Y cells treated with 1% BJ. Representatives images show the loss of the normal actin morphology characterized by stress fibres (arrows) *vs* a morphology characterized by shorter and contorted actin fibres. White bar = 20 µm.

The effect of BJ on cell migration was evaluated by modified Boyden chamber assay. Figure 7A shows the effect of BJ at 1% and 5% on migration of SH-SY5Y cells through gelatine-coated filters. After 24 hs of treatment with BJ, the percentage of migrating cells in comparison with untreated control cells was considerably reduced at all BJ concentrations (40±9% with 1% BJ and 20±8% with 5% BJ; P<0.01and P<0.001 *vs* untreated cultures, respectively).

It is known that flavonoids are able to interact with cytoskeleton proteins. Thus, we evaluated the consequence of BJ treatment on actin polymerization. The effect of BJ on actin polymerization and cell morphology was already evident in SH-SY5Y treated with 1% BJ for 24 hs (Fig. 7B). In these cells the actin fibers began to disappear and they were progressively concentrated at the cell periphery. In SH-SY5Y cells treated with 1% BJ stress fibers, well visible in untreated cells, were frequently substituted by shorter and contorted actin fibers (Fig. 7, arrows).

### BJ Inhibits FAK Phosphorylation via Inhibition of NCAM Activity and Decrease of their Association

**Figure 8:**
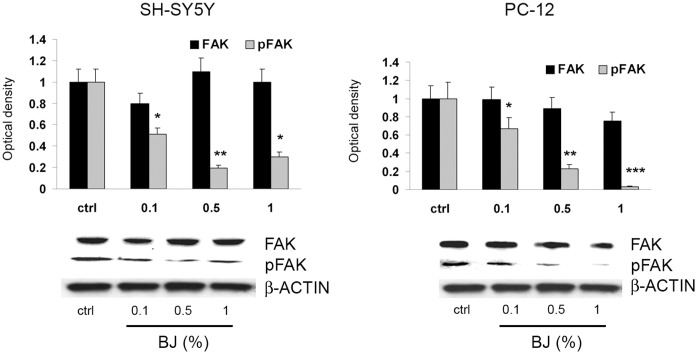
FAK and pFAK expression in SH-SY5Y and PC12 cells treated with BJ. Western blotting analysis of FAK and pFAK expression in SH-SY5Y and PC12 cells treated for 24 hs with increasing concentration of BJ (0.1%, 0.5% and 1%). Relative densitometric analyses of pFAK and FAK immunoreactive bands are presented in the histograms. Data (mean ± SEM of three experiments) were normalized to the values yielded for β-actin. *P<0.05, **P<0.01 and ***P<0.001 *vs* untreated cells. Note the significant reduction of pFAK by BJ.

The activation of Focal Adhesion Kinase (FAK) plays a key role in regulating focal adhesion complex formation, which in turn stimulates molecular events associated to adhesion and migration, including actin polymerization and stress fibres formation. The levels of phospho-FAK (pFAK) and total FAK were examined by immunoblot in SH-SY5Y and PC12 cells treated with BJ 0.1, 0.5 and 1% for 24 hs. In cells treated with BJ, no significant changes were seen in the expression of FAK, while we observed a significant decrease of the pFAK levels in both cell lines (Fig. 8).

**Figure 9:**
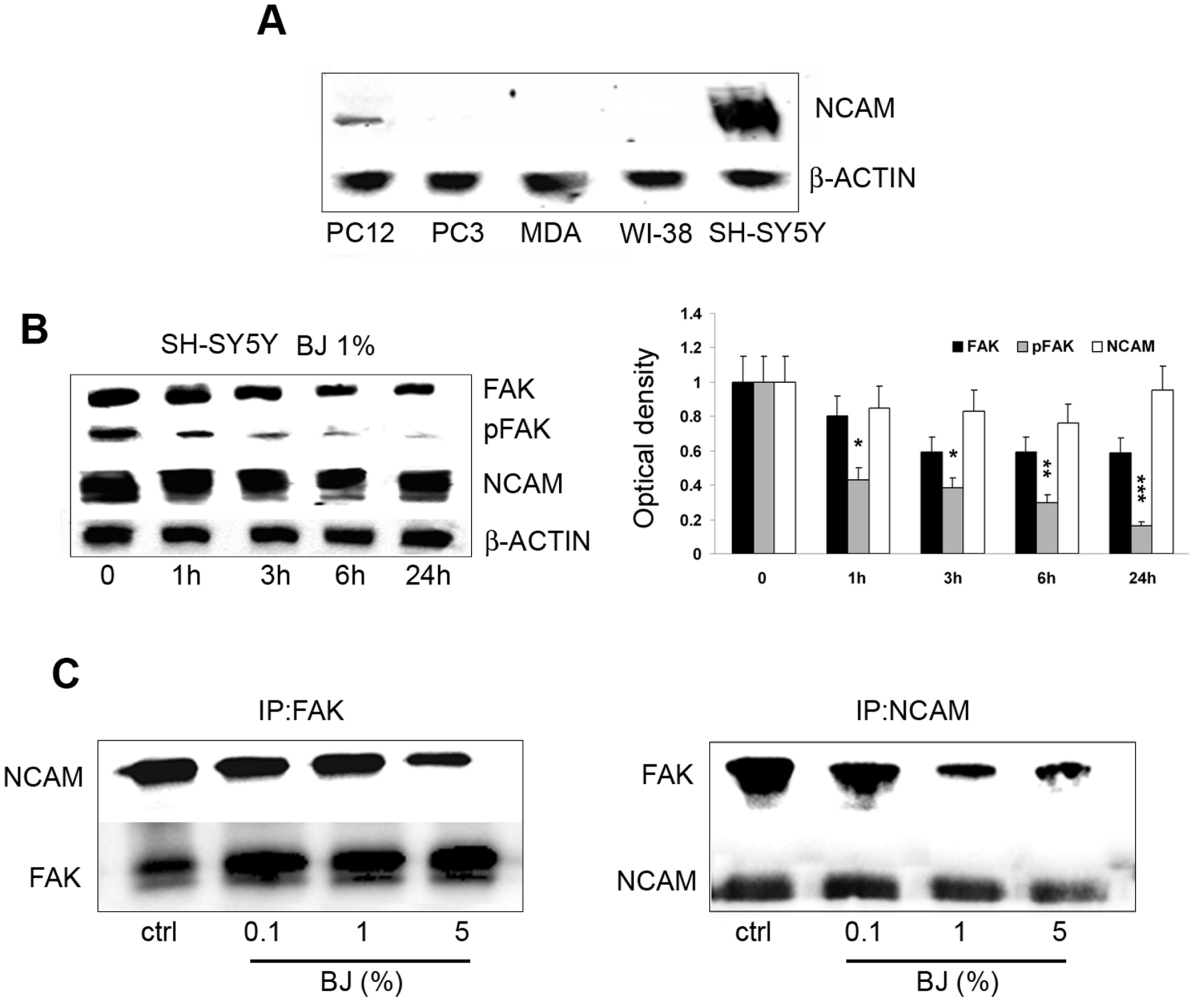
BJ effects on association between FAK and NCAM. (A) Western blotting analysis of NCAM basal expression in PC3, MDA-MB231, WI-38, PC-12 and SH-SY5Y cells. Note that NCAM is expressed only in both SH-SY5Y and PC12 cells. (B) Western blotting analysis of FAK, pFAK, and NCAM expression in SH-SY5Y cells treated with 1% BJ for different times (from 1 h to 24 hs). Relative densitometric analyses of immunoreactive bands normalized to the values for β-actin are presented in the lateral histogram (mean ± SEM of three experiments). *P<0.05, **P<0.01 and ***P<0.001 *vs* untreated cells. A slight reduction of NCAM within 6 hs of BJ incubation accompanied to a significant decrease of pFAK is shown. (C) Protein extracts from SH-SY5Y cells treated for 24 hs with 0.1–5% BJ were subjected to immunoprecipitation with FAK antibody (IP:FAK, left panel), or with NCAM antibody (IP:NCAM; right panel) and then analysed for the expression of both NCAM and FAK.

FAK phosphorylation requires the activation of the non-receptor tyrosine kinase fyn which interacts with and in turn is activated by neuronal cell adhesion molecule NCAM, stimulating cell migration [21]. It is known that in neuronal cells functional triggering of NCAM is a key event in cell migration [22]. In order to evaluate the involvement of NCAM in the anti-adhesive effect of BJ we examined its expression in the tumor cells line investigated in this study. As shown in figure 9A, NCAM is expressed only in SH-SY5Y and PC12 cells but not in the other tumor cells. Then we investigated the levels of pFAK, total FAK and NCAM in SH-SY5Y cells treated with 1% BJ in a time course experiment. Tests were executed only in the SH-SY5Y cells because these cells expressed higher levels of NCAM respect to PC12 cells. We observed an early marked decrease of pFAK expression starting from 1 h of treatment, suggesting a direct action of BJ in blocking FAK activation machinery (Fig. 9B). Moreover, we observed a progressive but slight reduction in NCAM expression within 6 hs after treatment with 1% BJ. However, after 24 hs NCAM levels returned similar to control levels.

Finally, we sought to verify whether BJ could interfere with interaction between FAK and NCAM. The co-immunoprecipitation assay performed on SH-SY5Y cells treated with increasing BJ concentrations (0.1–5%) for 24 hs demonstrated that although BJ did not modify the total expression of FAK and NCAM, it was able to reduce their reciprocal binding ability (Fig. 9C). A similar effect was observed also in cells exposed to BJ for 6 hs (results not shown).

## Discussion

Our study, for the first time, shows the antiproliferative and the anti-metastatic activity of BJ in *in vitro* models. In particular, BJ was able to inhibit the proliferation of several cancer cell lines with the maximal achievement in the SH-SY5Y neuroblastoma cells, in which caused arrest of the cell cycle accompanied with a marked reduction of cell adhesion mediated by an impairment of the actin network. Moreover BJ induced a decrease of cell migration determined by a down-regulation of FAK activity, with the involvement of cell adhesion molecules NCAM.

This study was firstly designed to investigate the chemo-preventive potential of BJ against the growth of several tumour cell lines. Treatment of SH-SY5Y, PC12, PC3 and MDA-MB-231 cells resulted in decrease of cell proliferation rate, showing the maximal growth inhibitory effect on the SH-SY5Y cells. The growth inhibitory effect of BJ is not mediated by a cytotoxic effect, because at the concentrations tested in this study we did not observe significant increase in cell death. The absence of DNA damage underlines the absence of genotoxicity by BJ. Of interest, we did not report cytotoxicity in non-tumoral cells such as the diploid fibroblasts WI-38 cells, highlighting the potential safety of the BJ. It is well known that cyclins play a pivotal role in controlling cell cycle checkpoints and that cyclin D1 is one of major proteins that enhance cell cycle progression through the G1-phase [23]. Investigating on the mechanism of cell growth inhibition, we found that BJ treatment of SH-SY5Y cells produced a block in the G1-phase of the cell cycle in a concentration and time-dependent manner, through a mechanism involving a reduction of cyclin D1 expression. As result, after 72 hs of exposure to the highest BJ concentration (5%), about 90% of cells were in the G1-phase, while there were not cells in G2/M-phase. Moreover, as indicated by cytofluorimetric and Western blot analyses, this alteration of the cell cycle was not coupled with an early activation of apoptotic machinery, excluding in this way the involvement of apoptosis as a mechanism of cell growth inhibition. These data suggest that, at concentrations which inhibited SH-SY5Y proliferation up to 65%, BJ acted in a cytostatic manner, without killing neither tumour cells nor the normal ones. In line with our results, several reports showed that many *Citrus* flavonoids exert their antiproliferative effects by modulating the cell cycle [24]. In particular, incubation of colon adenocarcinoma COLO 205 cells with tangeretin caused arrest of the cell cycle progression at G1 phase and growth inhibition [25], as well as naringin suppressed cell proliferation via G1-cell-cycle arrest in human bladder carcinoma 5637 cells [26]. Notably, tangeretin and nobiletin were further reported to inhibit the proliferation of both human breast cancer cell lines (MDA-MB-435 and MCF-7) and human colon cancer line (HT-29) by blocking cell cycle progression at the G1 phase without inducing apoptosis [27]. Cell cycle arrest devoid apoptosis has been described also in SH-SY5Y cells [28]. Moreover, our experiments demonstrated that BJ considerably altered the SH-SY5Y morphology within few hours of exposure, causing a marked reduction of cell adhesiveness. At 72 hs after treatment, nearly half of the SH-SY5Y cells were detached from the bottom of the wells. Likewise, about the 20% of vital PC12 cells were in suspension and floating in the medium. Therefore, through the impairment of tumour cell adhesive properties BJ may contribute in inhibiting cancer cell progression.

Our results also showed a relevant effect on actin filament assembly of SH-SY5Y just from 24 hs of treatment with 1% BJ. Accordingly, other authors have demonstrated that some flavonoids are able to bind actin interfering with its function in the cytoplasm and in the nucleus [29]. This effect appears important because actin microfilaments play many functional roles in cells, including maintaining of cellular morphology, cell adhesion and motility, cell cycle control, and cell death machinery, among many others [30], [31]. According to numerous studies indicating that actin cytoskeleton may acts as the central intracellular “motor” of cell motility and it is essential for most types of cell migration [32], [33], we observed a reduction of cell motility and cell adhesion of SH-SY5Y treated with BJ. Cell migration inhibition following the treatment with 1% BJ was accompanied by an impairment of SH-SY5Y adhesive ability on different physiologic substrates in a concentration-dependent manner. Similarly, adhesion assay on HUVEC monolayer showed that BJ starting from 0.25% significantly inhibited SH-SY5Y adhesion in comparison with untreated cells that efficiently adhered on HUVEC monolayer forming also cell clusters.

Cell adhesion turnover involves several tyrosine kinases and phosphatases, most of which are engaged in focal adhesion signalling pathways [34]. FAK is thought to be the main tyrosine kinase involved in focal adhesion complex and its activation plays an important role in interpreting signals from cell-cell and cell-matrix interactions. Therefore, activation of this kinase is crucial in the maintenance of an aggressive phenotype in cancer cells exposed to different extracellular stimuli [35]. In SH-SY5Y cells, FAK is rapidly phosphorylated upon cell adhesion leading to activation and phosphorylation of cytoskeleton-linked proteins that are responsible for signal propagation downstream of integrins [36]. Our results, showing a concentration dependent inhibition of FAK phosphorylation in response to BJ treatment, confirm the importance of FAK in regulating significantly the behaviour of SH-SY5Y cells. In fact we observed that hypo-phosphorylated FAK was associated to perturbation in actin polymerization and to a reduction in cell migration. On this subject, it has been demonstrated that flavonoids effects could depend on the extent to which they associate with cells, either by interactions at the membrane or by uptake into the cytosol. Moreover, an emerging concept is that flavonoids can modulate intracellular key signalling cascades. In this regard in a recent study it has been demonstrated that flavonoids interact with proteins involved in protein kinase (PK) and lipid kinase signalling cascades that are associated with cellular survival and apoptosis, such as those of phosphatidylinositol 3-kinase (PI-3K), Akt/PKB, tyrosine kinase, PKC, and mitogen-activated protein kinases (MAPKs). Flavonoids can bind to the ATP-binding sites of proteins (e.g., mitochondria ATPase, calcium membrane ATPase, PKA, PKC, and topoisomerase) or benzodiazepine-binding sites of receptors (e.g. GABA-A, adenosine receptors) and consequently alter their activities [37], [38]. Calcium ions act as second messengers and trigger kinase signalling including Akt, extracellular signal-regulated kinase (ERK) and c-Jun N-terminal kinase (JNK) signalling, while mitochondria mediate many regulatory pathways in which ATP is required. Hence, flavonoids can also modulate cellular signalling by maintaining calcium homeostasis and mitochondrial function [39]. FAK phosphorylation and focal adhesion assembly require the activation of the non-receptor tyrosine p59*^Fyn^* kinase which localizes in lipid raft in association with NCAM by which is in turn activated [40]. It is also known that functional triggering of NCAM leads to neural migration [41]. In particular, it has been demonstrated that NCAM140, localized in membrane compartments outside the lipid raft, binds fibroblast growth factor receptor (FGFR), but in consequence of down-modulation of the cell-cell adhesion molecule E-cadherin, it migrates towards the lipid raft. This condition is a hallmark of epithelial-mesenchimal transition (EMT) in the progression from epithelial tumours to invasive and metastatic cancers [21]. It is known that cancerous cells often exhibit impaired intercellular adhesion as a prerequisite for their invasive behaviour. This can be associated in some solid tumors, including small cell carcinoma, neuroblastoma and rhabdomyosarcoma with an over-expression of selected cell adhesion molecules, including NCAM 42,43. Of interest, in our study we observed a slight modulation of NCAM expression in SH-SY5Y cells treated with 1% BJ within 6 hs of incubation. Evidence that BJ inhibits both pFAK and NCAM, even with different degrees, suggests its ability to hinders the association between these two important molecules.

Different studies have demonstrated the ability of single bioactive molecules found in *Citrus* fruit to inhibit cancerogenesis both *in vitro* and *in vivo*, as well as other investigations have suggested their capacity to hinder invasion and metastasis [24], [44], [45]. However, few studies have focused on the biological activity of *Citrus* juices. It has been reported that commercial Satsuma mandarin (*Citrus unshiu* Marc.) juice, especially with addition of higher amounts of flavonoids such as β-cryptoxanthin and hesperidin, reduced both chemically-induced rat colon carcinogenesis [46] and lung cancer formations [47], as well as So et al. [48] documented that concentrated orange juice decreased the chemically-induced mammary tumors burden in rats. Some years later also Miyagi et al. [49] indicated that orange juice inhibited azoxymethane (AOM)-induced colon cancer in rats.

This study for the first time demonstrated the anticancer potential of BJ, a finding of great relevance in health-keeping field in view of the fact that the pharmacological effects observed in our experiments were obtained using a natural juice easily obtained by hand-squeezed bergamot fruits. In line with other authors [50], [51] we feel that the complex mixtures of phytochemicals present in fruits and vegetables could be more effective than their individual constituents in preventing cancer through both additive and synergistic effects. Hence the importance to study potential anticancer activity of fruits and vegetables by using whole extracts containing all phytochemicals [51].

Extrapolation of preclinical results through an automatic transfer of data for human use cannot be operated. Moreover, until now pharmacokinetic studies or appropriately designed clinical trials to assess the bioavailability of bioactive molecules contained in BJ have not been performed. However, several commercial herbal preparations of Citrus bioflavonoids exist. To our best knowledge, the most common dosage of naringin and eriocitrin when combined in a formula with other supplements is of 100 mg. Furthermore, commercial preparations made from bergamot polyphenolic extract contain 250 mg of flavonoids such as neoeriocitrin, naringin and neohesperidin that represent the major constituents of our BJ. On the basis of BJ flavonoidic content, we hypotized that the concentrations required for BJ to achieve the antitumor effects *in vitro* are also achievable *in vivo*. Indeed, the anti-invasive/anti-migration activity exerted at the 1% concentration of BJ may be reached by assuming less than 1 liter of BJ; higher amounts (up to 5 liters) of BJ is required for a potential antiproliferative effect obtained with the 5% BJ. In addition, we suppose that the conversion of natural BJ in a commercial product would better achieve adequate clinical concentrations, allowing its use as supplement in the healthy field.

In summary, our findings demonstrated the ability of BJ to affect important tumoral activities of cancer cells. In particular, the loss of adhesive morphology of SH-SY5Y cells and the growth reduction following BJ treatment suggested that this compound is able to interfere with molecular and cellular processes involved in cytoskeleton reorganization, actin remodelling and cell cycle progression. Because these cellular processes are peculiar of the malignant phenotypes, our findings suggest a promising role of BJ as supplement or adjuvant treatment aimed to support conventional therapy. This study represents the basis for further investigations on the potential use of BJ in oncology field, although additional studies on different cancer models are necessary to consolidate our data.

## Materials and Methods

### Bergamot Juice

The fruits of *Citrus Bergamia* Risso and Poiteau were collected from a cultivation located in Bovalino (Reggio Calabria, Italy). Ms. Oliva Francesca is the owner of private land and the bergamot fruits were a generous gift of her. The fruits were immediately hand-squeezed and small aliquots of BJ were collected in sterile tubes and stored at −20°C. Afterwards, BJ was defrosted, adjusted pH to 7.4, filtered (progressively up to use filters with 0.22 µm pore size), and diluted in culture media until the desired concentrations just prior to use.

### Analysis of Flavonoids by RP-HPLC

The BJ was analyzed without any pre-treatment: the juice was centrifuged and then filtered on Acrodisc filter 0.22 µm (Sigma-Aldrich, St. Louis, MO). The sample was analyzed for five times by RP-HPLC. The HPLC analyses were carried out on an Ascentis Express C18, 5 cm×4.6 mm i.d. with particle size of 2.7 µm (Supelco, Bellefonte, PA). The injection volume was 2 µL: mobile phase consisted of water/formic acid (99.9∶0.1) (solvent A) and water/acetonitrile/2-propanol/formic acid (39.9∶20:40∶0.1 v/v) (solvent B). The linear gradient profile was as follows: 0–3 min, 10% B, 3–43 min, 10–42% B, 43–47 min, 42% B, 47–57 min, 100% B, 57–60 min, 10% B. Flow-rate was 0.8 mL/min. Data were acquired using a photodiode array detector in the range 190–370 nm and the chromatograms were extracted at 283 and 325 nm. Time constant was 0.64 s and sample frequency 1.5625 Hz. The Mass Spectrometer (MS) acquisition was performed using ESI, in negative mode, under the following conditions: mass spectral range, *m/z* 250–700; interval, 0.5 s; scan speed, 938 amu/s; nebulizing gas (N_2_) flow, 1.5 L/min; ESI temperature, 350°C; heat block, 300°C; DL (desolvation line) temperature, 300°C; DL, voltage −34V; probe voltage, +4.5 kV; Qarray voltage, 1.0 V and detection gain, 1.05 kV. To quantify the content of flavonoids present in the BJ, calibration curve was constructed by using naringin as reference material. Five different concentrations of naringin, in the 100–0.1 mg/L concentration range, were prepared by diluting a stock solution of about 1000 mg/L, using methanol as a solvent, and analyzed for five times by HPLC under the same chromatographic conditions optimized for the juice sample. Limit of detection (LOD) and limit of quantification (LOQ) values were calculated as reported [52]. Calibration curve of naringin was taken into account for the determination of the flavanone-*O*-glycosides content in the bergamot juice.

HPLC separation was carried out on a Shimadzu system equipped with two LC 10 AD *Vp* pumps, a SCL-10-A*vp* controller, a DGU-14A degasser, an SPD-M10 A*vp* UV detector and a Shimadzu LC-MS-2020. Data acquisition was performed by Shimadzu LC solution software ver 3.3. Naringin, neoeriocitrin, eriocitrin and neohesperidin were purchased from Sigma-Aldrich (Milan, Italy), as well as water, methanol, acetonitrile and 2-propanol. All solvents were HPLC-grade. Since the pH of the bergamot juice is roughly 3.5–3.8, all the mobile phases have been adjusted to the appropriated pH value (pH 3) with formic acid in order to suppress the ionization of the phenolic groups [52]. No specific permits were required to describe the composition of BJ.

### Cell Culture and Cell Growth Analysis

Experiments were carried out by using the tumour derived SH-SY5Y human neuroblastoma (NB) cell line, human breast cancer cell line MDA-MB-231, human prostatic cell line PC3, PC12 cell line, derived from a pheochromocytoma of the rat adrenal medulla, and fibroblast WI-38, derived from normal embryonic lung tissue as a control. All cell lines were obtained originally from ATCC (Rockville, MD, USA). SH-SY5Y were grown in RPMI supplemented with heat-inactivated fetal bovine serum (FBS 10% v/v), 2 mM glutamine, penicillin (100 lU/ml), and streptomycin (100 µg/ml). PC12 were maintained in RPMI supplemented with heat-inactivated fetal bovine (5%) and horse (10%) serum. PC3 were cultured in COON’S supplemented with 10% FBS, penicillin-streptomycin. MDA-MB-231 and Wi-38 cell lines were grown in DMEM supplemented with 10% FBS, glutamine and penicillin-streptomycin. All reagents for cell cultures were from Gibco (Life Technologies, Monza, Italy). To determine cell proliferation, the MTT test were performed as reported [53] with modifications. Indeed, in SH-SY5Y and PC12 cells BJ incubation caused a dramatic detachments from the bottom of the wells, that did not allow us to perform the classic protocol. The cells were seeded onto 6-well plates (100×10^3^ cells/well) and 24 hs later the media was replaced with fresh medium (untreated cultures) or with medium containing increased dilution of BJ, ranging from 0.5 to 5%. After 24, 48 and 72 hs of incubation, all the cells present in a single well were collected in clean tubes (floating cells by transferring of the culture medium and attached cells by trypsinization) and centrifuged. Then, the supernatants were replaced with 1 ml fresh media without phenol red containing 0.5 mg/mL of 3-(4,5-dimethylthiazole-2-yl)-2,5-diphenyltetrazolium bromide (MTT; Sigma-Aldrich; Milan, Italy). The samples were returned in the incubator for 4 hs and gently shaken occasionally. Then, crystals of formazan (MTT metabolic product) were solubilized by 1 ml ethanol/dimethyl sulfoxide (DMSO) 1∶1 lysis buffer and spectrophotometrically quantified (Shimadzu UV 1601 Spectrophotometer, Kyoto, Japan) at a wavelength of 570 nm with reference at a wavelength of 695 nm. MTT test was also performed using the PC3, MDA-MB-231 and WI-38 cell lines. The cultures were seeded onto 96 well plates (5×10^3^ cells/well) and incubated with the BJ as described above. Then, the tests were carried out according to the instructions. Differences in cell growth were measured as a percentage of growth rates of treated cells compared to untreated cultures. Cell growth was also detected by the cell counted assay. Briefly, SH-SY5Y cells were seeded and treated in the same experimental condition described above. Afterwards, cells were harvested, centrifuged and resuspended in a known amount of culture medium. Aliquots of cell suspensions were put in a Neubauer hemocytometric chamber and cells where count by a common optical microscope.

### Cytotoxicity Assays

Possible cytotoxicity of BJ was evaluated in terms of cell death and assessed by the trypan blue dye exclusion test, as reported [54]. The experiments were carried out on SH-SY5Y, PC12 and WI-38 cells. Further, in order to determine the potential genotoxic effect of BJ, the comet assay was performed as reported [55]. In brief, at the end of BJ exposure, both treated and untreated SH-SY5Y cells were collected, washed with ice-cold PBS and 10 µl of cell-suspension (1×10^6^ cells/ml) were dissolved in Low Melting Point (LMP) Agarose and spread on agarose-precoated microscope slides. The cells were lysed in high salt and detergent overnight at 4°C and then placed in a horizontal electrophoresis box. Subsequently, the cells were exposed to alkaline conditions for 20 min to allow for DNA unwinding and expression of alkali-labile sites. To electrophorese the DNA, an electric current of 25 V (0.86 V/cm) and 300 mA was applied for 20 min. Then, the slides were neutralized, stained with ethidium bromide (10 mg/ml) and analyzed using a DM IRB fluorescence microscope at a 400× magnification (Leica Microsystems Heidelberg, Mannheim, Germany), equipped with a digital camera (Canon Power Shot S50, Milan, Italy). Etoposide (50 µM; Sigma-Aldrich) was used to induces DNA damage. For each sample, images of at least 100 randomly selected nuclei were acquired and submitted to the Comet Assay Software Project (CASP) Lab automated image analysis system. The considered parameters were tail length (TL), percentage of DNA in the head (HDNA %), percentage of DNA in the tail (TDNA%), tail moment (TM) and Olive tail moment (OTM).

### Cytofluorimetric Analysis

The Annexin V fluorescein isothiocyanate (FITC) staining was performed to detect apoptosis. Briefly, SH-SY5Y cells were seeded in 6-well plates (100×10^3^ cell/well) and 24 hs later were treated with the BJ (1–5%). After 24, 48 or 72 hs of incubation, the cells were harvested, washed, centrifuged and suspended in 100 µl HEPES buffer (10 mM HEPES, 150 mM NaCl, 1.8 mM CaCl_2_, 1 mM MgCl_2_ and 5 mM KCl, pH 7.4). Then, 5 µl of Annexin-V FITC (Becton Dickinson, Franklin Lakes, NJ) were added in each samples, gently vortexed and incubated for 15 min at room temperature in the darkness. Samples were washed in 500 µl of cold PBS and 5 µg/ml TO-PRO (Life Technologies Corporation, Grand Island, NY) was added to distinguish the necrotic cells. Finally, specimens were run on flow cytometer. Cultures treated with etoposide (50 µM) were used as positive control of apoptosis.

Cytofluorimetric studies were used also to check the progression of cells trough the cell cycle. SH-SY5Y were seeded and treated in the same experimental condition described above. According to the CycleTEST™ PLUS DNA Reagent Kit instructions (Becton Dickinson), the cells were collected by trypsinization, centrifuged, washed in a buffer solution containing sodium citrate, sucrose, DMSO, and fixed in cold 70% ethanol at 4°C for at least 2 hs. Then, the cells were centrifuged, washed in PBS and resuspended in 250 µL of Solution A (trypsin in a spermine tetrahydrochloride detergent buffer) for 10 min at room temperature prior to add 200 µL of Solution B (trypsin inhibitor and ribonuclease A in citrate stabilizing buffer with spermine tetrahydrochloride) for further 10 min. Finally, 200 µL of cold Solution C (propidium iodide and spermine tetrahydrochloride in citrate stabilizing buffer) were added, gently mixed and incubated overnight in the refrigerator. In all cytofluorimetric studies five independent sets of 20.000 events were collected for each condition.

### Migration Assay

Cell migration was quantified by a modified Boyden chamber assay [56]. Briefly the surface of the PVP free 8 µm polycarbonate filters (Nucleopore, Concorezzo, Milan, Italy) was coated with 0.01% gelatine w/v, rinsed once with PBS and then placed in contact with the lower chamber of the filter where the media containing low serum concentration (2% FBS), which allows cell survival but not cell proliferation, was added. Next, cells (1×10^5^ per chamber) were trypsinized, washed twice with PBS, rinsed in complete medium and incubated at 37°C for 30 min to reconstitute the membrane structures. Then cells were added to the top of each chamber in medium FBS deprived in presence of different concentration of BJ (1 and 5%). Cells were allowed to migrate through the coated filters for 6 hs. The cells attached on the lower membrane surfaces were stained with 0.5% crystal violet w/v in 20% methanol w/v for 20 min at room temperature. Cells were counted at 400× magnification in standard optical microscopy and the average number of cells per field in 5 random fields was recorded. Triplicate filters were used and the experiments were repeated three times.

### Cell Adhesion Assay

First, cell suspensions of SH-SY5Y or PC12 were seeded onto 6-well plates (100×10^3^ cells/well) and treated with various concentrations of BJ for different times of incubation. The morphology of SH-SY5Y cultures was photographed after 2 hs of incubation with BJ by using an inverted light microscope. Following 24, 48 and 72 h of exposure to BJ (1–5%), the cells (SH-SY5Y or PC12) were detached by gentle agitation, washed off with culture media, collected and counted in Neubauer hemocytometric chamber in presence of trypan blue dye to distinguish between live and dead cells in suspension. Adherent cells were harvested by trypsinization and counted as well.

The ability of SH-SY5Y cells to adhere to matrices simulating physiological substrates in presence of BJ was further assessed by a second step of experiments. Twenty-four-well plates were pre-coated with fibronectin (1 µg/ml), matrigel (1 µg/ml) and type I collagen (5 µg/ml), maintained to 4°C overnight and then washed twice with a wash solution (0.1% BSA DMEM), and blocked with 0.5% BSA. Adherent cells were fixed with cold methanol, washed with PBS and air-dried. After staining with crystal violet 0.5% w/v for 15 min, cells were lysed with a solution containing 2% sodium dodecyl sulphate (SDS) w/v, 50% methanol in water for 1 h with gentle agitation. Lysed cells were transferred to 96-well plates and absorbance was measured at 595 nm. The rate of cell adhesion was calculated considering control absorbance as 100%.

### Cell-cell Adhesion Assay

To prepare endothelial monolayers, human umbilical vein endothelial cells (HUVEC; Lonza, Basel, Switzerland), were resuspended in endothelial cell growth medium 199 (M199) containing 2 mM glutamine, 100 µg/ml penicillin/streptomycin, 100 µg/ml heparin, 30 µg/ml endothelial growth factors and supplemented with 15% FBS. HUVEC were seeded (2×10^5^ cells per well) in 48-well plates 24 hs before adhesion assay and incubated at 37°C in 5% CO_2_. SH-SY5Y plates were pre-treated with various concentrations of BJ for 24 hs (0.25–5%) and pellet cells obtained by trypsin detachment were purified by centrifugation, re-suspended in warmed adhesion buffer (0.5% BSA in PBS) at 8×10^5^ cells/ml and incubated for 60 min to room temperature. After washing with warm adhesion buffer, HUVEC monolayers were fixed with formaldehyde (3.7% in PBS), washed twice and blocked with 10 mM EDTA for 60 min. Then SH-SY5Y were seeded on HUVEC monolayer previously washed with adhesion buffer and leaved to settle for 45 min. To allow counting of adherent cells, not adherent cells were removed by washing with PBS and adherent cells photographed immediately choosing at least five different fields at random. The acquired images were quantified by an Image Pro-plus v 4.5 analysis system.

### Western Blotting and Immunoprecipitation

Total cellular lysates were prepared by resuspending SH-SY5Y treated and untreated with BJ for different times in a lysis buffer containing 1% TritonX100 v/v, 0.1% SDS w/v, 2 mM CaCl_2_, 100 µg/ml phenylmethyl sulphonyl fluoride. The protein content was determined using the Protein Assay Kit 2 (Bio-Rad Laboratory, Hercules, CA, USA) and protein extracts (60 µg/lane) of SH-SY5Y were electrophoresed in 8% SDS-polyacrylamide gel and then electro-transferred to nitrocellulose membrane (Protram, Whatman, Germany). The membranes were then incubated with: a 1∶1500 dilution mouse monoclonal antibodies against Cyclin D1 (Santa Cruz Biotechnology Inc., Santa Cruz, CA, USA), a mouse monoclonal anti-caspase-9 diluted 1∶1000 (Cell Signaling Technology Inc., Danvers, MA, USA), a rabbit polyclonal anti-p53 diluted 1∶100 (Santa Cruz Biotechnology Inc.), a rabbit polyclonal anti-caspase-3 (AbCam, UK) or β-actin (Cell Signaling Inc.) both diluted 1∶1000, a mouse monoclonal anti-FAK or anti-pFAK or anti-NCAM, all diluted 1∶200 (Santa Cruz Biotechnology Inc.). Then membranes were incubated with specific horseradish peroxidase-conjugated secondary antibodies. Protein bands were visualised using a chemiluminescent detection system (Thermo Scientific, Rockford, USA).

For FAK immunoprecipitation, lysates with the same amount of protein in the same volume were incubated overnight at 4°C with anti-FAK antibody, and protein-A-Agarose beads (Santa Cruz Biotechnology Inc.) were then added for a further 2 hs. After washing once with the cold lysis buffer and four-times with ice-cold PBS, the immunoprecipitates were then processed for detecting co-immunoprecipitated NCAM as previously described in Western blotting section. A parallel NCAM immunoprecipitation was realized incubating lysates with protein agarose beads adsorbing an anti-NCAM antibody and processing immunoprecipitates to visualize co-immunoprecipitated FAK proteins.

### Statistical Analysis

Data were expressed as mean ± S.E.M. and statistically evaluated for differences using one-way analysis of variance (ANOVA), followed by Turkey-Kramer multiple comparison test (GrafPAD Software for Science, San Diego, CA). P values less than or equal to 0.05 were considered significant.
