# Correction: Mechanisms Underlying the Anti-Tumoral Effects of *Citrus bergamia* Juice

**DOI:** 10.1371/journal.pone.0206630

**Published:** 2018-10-25

**Authors:** Simona Delle Monache, Patrizia Sanità, Elena Trapasso, Maria Rita Ursino, Paola Dugo, Marina Russo, Nadia Ferlazzo, Gioacchino Calapai, Adriano Angelucci, Michele Navarra

The authors would like to correct an image duplication error in Fig 8. In Fig 8, the β-actin Western blot for SH-SY5Y cells was included in the PC-12 panel in error. The updated Fig 8, below, includes the correct PC-12 β-actin blot from the original experiment. The authors confirm that the densitometry data shown in the bar graph for PC-12 cells, both in the original and corrected versions of the figure, were obtained using the correct control blot. Densitometric analysis was conducted using ImageJ software (see https://imagej.nih.gov/ij/docs/menus/analyze.html). The raw densitometry data underlying the graphs in Fig 8 are no longer available.

**Fig 8:**
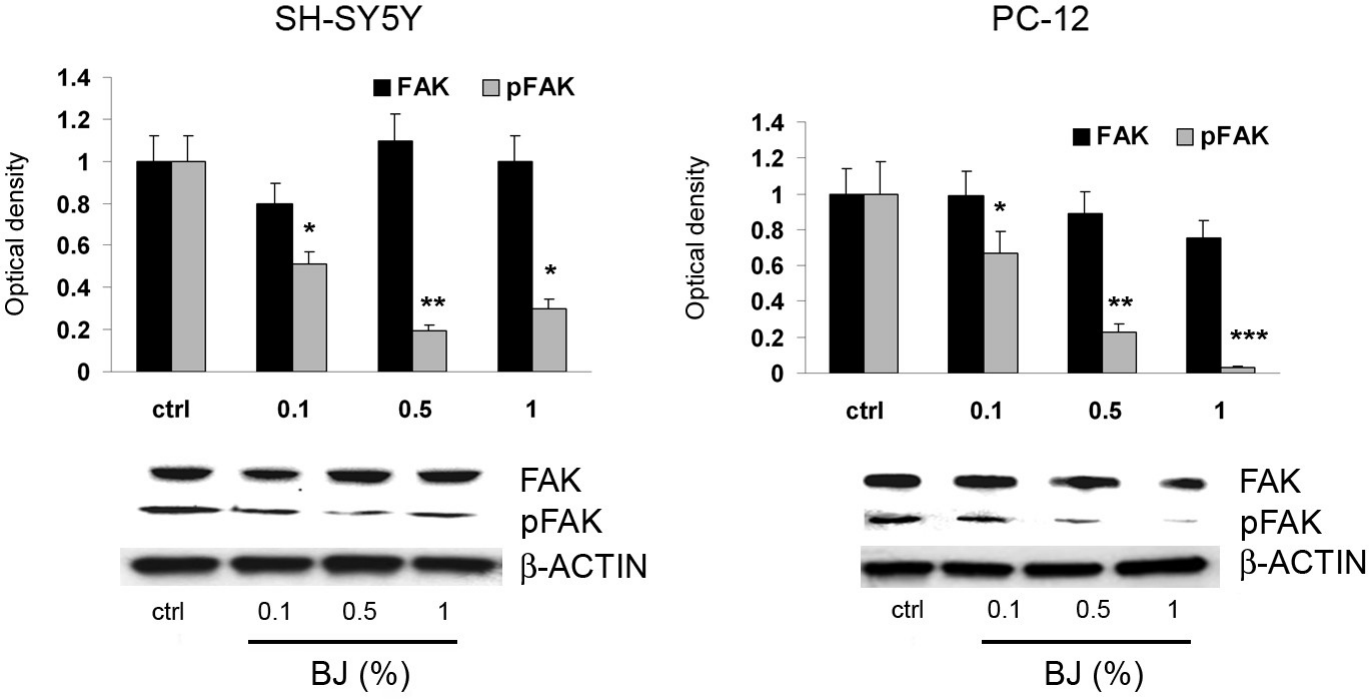
FAK and pFAK expression in SH-SY5Y and PC12 cells treated with BJ. Western blotting analysis of FAK and pFAK expression in SH-SY5Y and PC12 cells treated for 24 hrs with increasing concentration of BJ (0.1%, 0.5% and 1%). Relative densitometric analyses of pFAK and FAK immunoreactive bands are presented in the histograms. Data (mean ± SEM of three experiments) were normalized to the values yielded for β-actin. *P<0.05, **P<0.01 and ***P<0.001 *vs* untreated cells. Note the significant reduction of pFAK by BJ.

The original Western blots from which the Fig 8 panels were generated are no longer available, and so the authors repeated the Western blot analyses to confirm the Fig 8 results. The new Western blot results and the original uncropped blot images for these experiments are in S1 and S2 Files.

A member of *PLOS ONE*’s Editorial Board has reviewed the replication data provided and confirmed that they support the published findings.

In addition, the authors clarify that in Fig 9, the blot images presented for different antigens are from distinct Western blot experiments using the same protein samples; the blot images were grouped within the respective panels in preparing the figure.

## Supporting information

S1 File Results from replicate Western blot experiments. (TIF)

S2 File Raw, uncropped blots for replicate experiments. (TIF)
