# Supplementary figures and images for: Correction: Mechanisms Underlying the Anti-Tumoral Effects of Citrus bergamia Juice
Source: PLoS One. 2018 Oct 25;13(10):e0206630. doi: 10.1371/journal.pone.0206630 (PMC6201950; doi:10.1371/journal.pone.0206630)

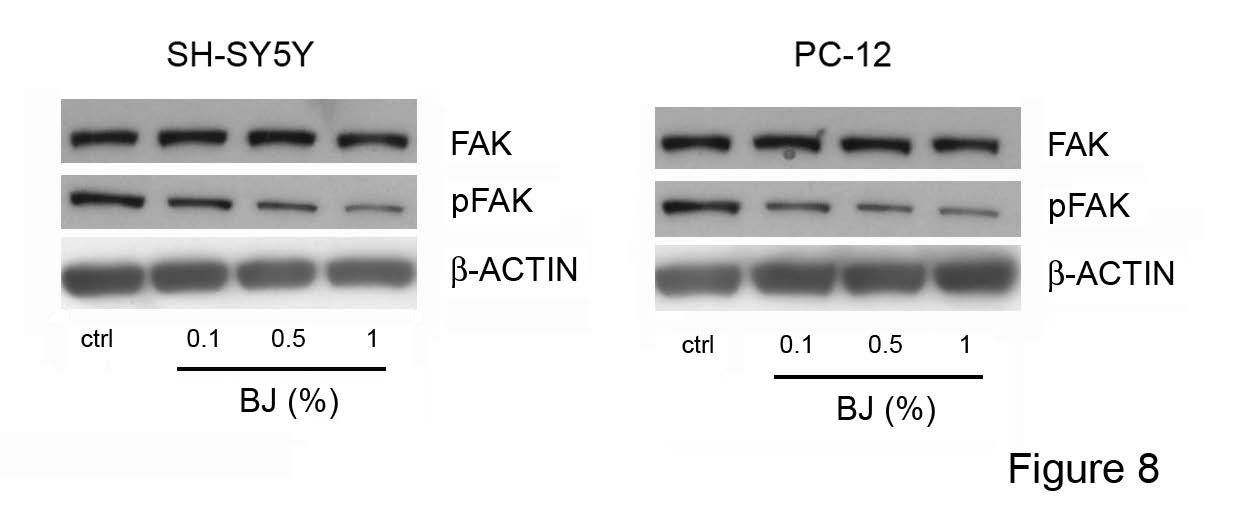

Supplement: S1 File — (TIF) [file pone.0206630.s001.tif]

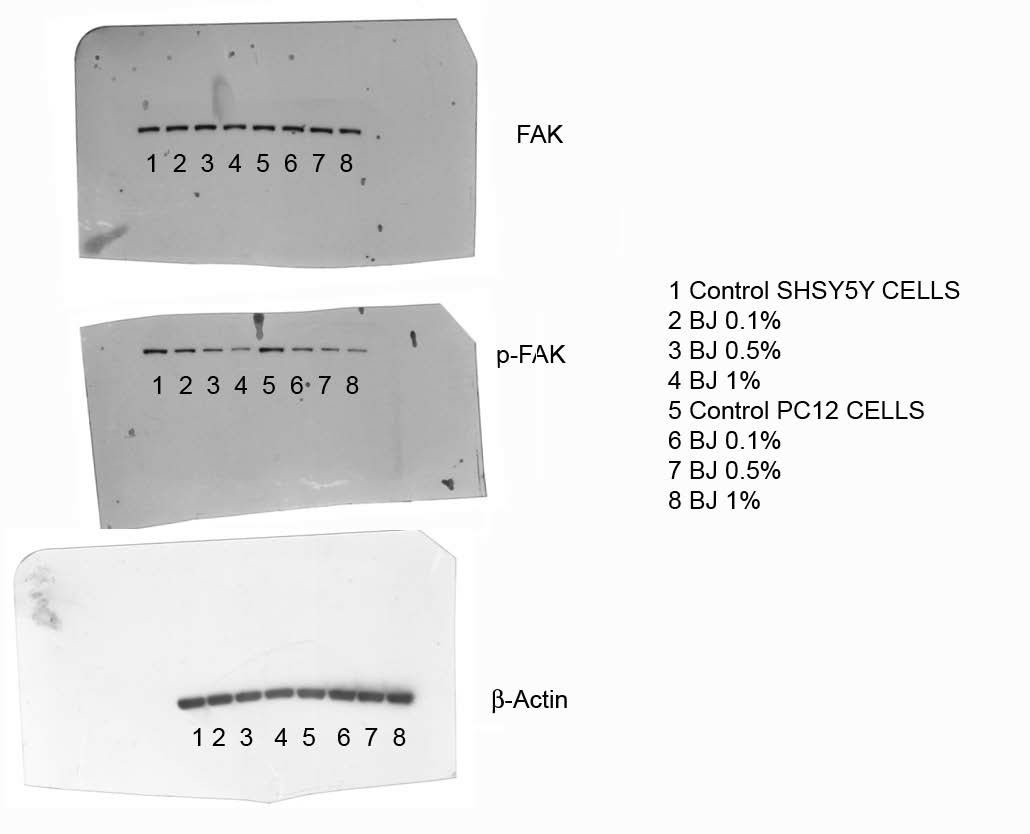

Supplement: S2 File — (TIF) [file pone.0206630.s002.tif]
